# Using More Equitable Integrated Care Programs to Reduce Fragmentation in Home Care

**DOI:** 10.5334/ijic.6553

**Published:** 2022-09-13

**Authors:** Krystal Kehoe MacLeod

**Affiliations:** 1 Carleton University, Ottawa, Ontario, Canada; 2 Centre for Research in Integrated Care, University of New Brunswick Saint John, New Brunswick, Canada

**Keywords:** equity, fragmentation, home care, integrated care, neoliberalism, older adults

## Abstract

Integrated care programs have been developed to deliver care across providers, settings, and support systems to meet the needs of community-dwelling older adults. This research asks how these programs are being used to combat issues of fragmentation in a home care sector fundamentally reshaped by neoliberalism. Data was collected through 118 key informant interviews in five Canadian integrated care programs and interpreted using a thematic analysis informed by Feminist Political Economy. This dissertation argues that integrated care programs are most useful as a policy solution to fragmented home care when they adopt policy techniques that promote equitable processes and outcomes.

## Introduction

Home care is the delivery of health and social care in private residential dwellings to help people live as independently as possible in the community [1]. The restructuring of the home care sector under neoliberalism since the early 1990s has revolved around establishing more efficient practices in response to issues of fragmentation [2]. Neoliberalism is the dominant political and economic ideology, governance structure, and policy toolkit that promotes the use of business solutions to public policy problems [3]. Fragmentation occurs when care systems and providers “function in silos” [4] causing gaps and duplication in services to appear within and across settings [5]. Fragmented home care results in a care system that is insufficiently equipped to meet the needs of older adults [6,7] and their paid and unpaid care providers [8], leading to worse health outcomes for all [5]. Integrated care programs that “deliver care that is coordinated across carers, care sites, and support systems; continuous over time and between visits; tailored to clients’ expressed needs and preferences; and based on shared responsibility for optimizing health outcomes” [9], have been positioned by decision-makers as a promising solution to fragmentation [10,11]. Researchers argue that better integrated care can contribute to improved health outcomes for clients [12,13]; increase older persons’ satisfaction with care [14,15,16]; increase access to care [17]; fulfill clients’ care needs [18,19]; and improve clients’ quality of life [20,21,22], all while simultaneously reducing the burden of healthcare costs on governments [23].

Based at Carleton University in Ottawa, Canada, this dissertation sought to understand the complexities of using integrated care within the current neoliberal system by asking the research question: how are integrated care programs being used as a tool to combat issues of fragmentation in the home care sector? This dissertation argued that integrated care programs are most useful as a policy solution to fragmented home care when they promote equitable processes and outcomes. Although the increasing importance of integration to health and social care delivery systems has not escaped scholarly and policy attention, the significance of using integrated care programs as a policy solution within a neoliberal context remains understudied. This dissertation makes a unique contribution in its critical analysis of the complexities of integrating care in a home care sector that has been profoundly reshaped by neoliberalism.

## Methodology

Purposive sampling [24] was used to select five programs across Canada that provided home care to older adults living in private residential dwellings using “integration” or “integrated care” as a guiding principle of service delivery. See Table 1 for a summary of program characteristics.

Program administrators, paid care workers, unpaid carers, and clients in five programs were invited to collaborate in semi-structured, in-person interviews (N = 118) in 2013. After obtaining informed consent, interviews lasted between 23 minutes and 1 hour 55 minutes. Digital recording of interviews enabled full data capture. Interviewing ceased when the point of saturation was reached [25,26]. Interviews were transcribed verbatim and analyzed using thematic analysis [27,28,29] where codes were analyzed and collated to form four overarching themes related to how the programs met clients’ and carers’ expressed needs. These themes were interpreted using a feminist political economy theoretical framework [30] to situate the impacts of integrated care programs within the larger political, economic, and social contexts that shaped the relationships and experiences of research participants.

**Table 1:**
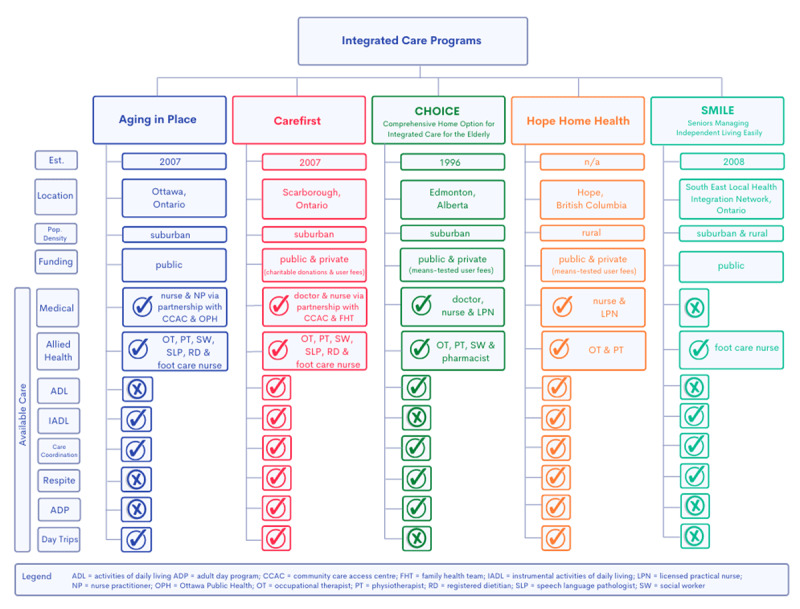
Summary of the key elements of the integrated care programs studied.

## Findings

Drawing on interview data in the four areas of expressed need identified by research participants: continuity of care, social inclusion, collaborative decision-making, communication/joint working, this dissertation found that programs’ use of policy techniques that prioritized equitable processes and outcomes offered a promising solution to fragmented home care. Policy techniques such as: providing care without user fees, supporting care workers, making time for social support, and facilitating collective forums for carers, distributed resources more equitably between, and among, clients, unpaid carers, and paid workers, as well as worked to reduce power disparities within, and across, groups. In integrated care programs using equity-promoting techniques, clients from marginalized groups were less likely to need to look outside the program to get their needs met; care workers reported more collaborative workplace relationships with better communication among workers and with management; and there was a more seamless provision of services for the benefit of clients, unpaid carers, and paid workers – all contributing to less fragmentation in home care delivery.

Conversely, when programs adopted policy techniques aligned with the neoliberal tendency to seek market-oriented solutions to issues of fragmentation through enhanced efficiency, findings showed that the needs of clients, unpaid carers, or paid care workers in positions of power and privilege were frequently prioritized over the needs of less powerful groups. Policy techniques including: service cuts, contracting out, task-shifting, lean staffing levels, work intensification, self-responsibilization, independent contracting agreements, flexible funding arrangements, and prescriptive care plans, were found to contribute to increased economic, race/ethnicity and/or gender inequality among program participants which, in turn, perpetuated fragmentated care provision as subsets of clients were unable to access care and care workers experienced workplaces marred by racism, marginalization, and precarious working conditions.

## Implications

Integrated care programs have been fundamentally shaped by the neoliberal context in which they operate in ways that have resulted in fragmentation, inefficiency, inequality, and equity being inextricably linked. When efficiency and equity goals are played against each other [31], it has resulted in efficiency being emphasized over equity. However, this dissertation showed that when neoliberal policy solutions aimed at enhancing efficiency are prescribed to redress fragmentation, in many cases these solutions perpetuated the fragmentation they viewed as inefficient. This is an example of one of neoliberalism’s “messy actualities” or “counter-tendencies” [32]. Yet, integrated care programs also demonstrated the ability to disrupt the “common sense understanding in society” [33] that increasing efficiency will reduce fragmentation. When integrated care programs prioritized equitable outcomes, they helped reduce fragmentation in home care access and service delivery. Neoliberal tendencies make it difficult to adopt a synergistic approach that reconciles equity with efficiency but there is much to be gained by clients and care providers if integrated care programs would attempt to do this. In the meantime, intentionally selecting policy tools that create conditions of work and care where the costs and benefits of program involvement are more equitably distributed within, and among, clients and care workers is a promising step towards reducing fragmentation in care access and delivery for older adults aging in place.

## IRB

- Research Ethics Board at Carleton University (Project #13-0785)
- CapitalCare’s Research Facilitation Committee (2013/02/15)
- Fraser Health Research Ethics Board (FHREB 2013-015)
